# Evidence Synthesis of Digital Interventions to Mitigate the Negative Impact of the COVID-19 Pandemic on Public Mental Health: Rapid Meta-review

**DOI:** 10.2196/23365

**Published:** 2021-03-10

**Authors:** Christian Rauschenberg, Anita Schick, Dusan Hirjak, Andreas Seidler, Isabell Paetzold, Christian Apfelbacher, Steffi G Riedel-Heller, Ulrich Reininghaus

**Affiliations:** 1 Department of Public Mental Health Central Institute of Mental Health Medical Faculty Mannheim, Heidelberg University Mannheim Germany; 2 Department of Psychiatry and Neuropsychology School of Mental Health and Neuroscience Maastricht University Maastricht Netherlands; 3 Department of Psychiatry and Psychotherapy Central Institute of Mental Health Medical Faculty Mannheim, Heidelberg University Mannheim Germany; 4 Institute and Policlinic of Occupational and Social Medicine Faculty of Medicine Carl Gustav Carus Technische Universität Dresden Dresden Germany; 5 Institute of Social Medicine and Health Systems Research Otto von Guericke University Magdeburg Magdeburg Germany; 6 Institute of Social Medicine, Occupational Health and Public Health University of Leipzig Leipzig Germany; 7 Centre for Epidemiology and Public Health, Health Service and Population Research Department Institute of Psychiatry, Psychology & Neuroscience King’s College London London United Kingdom; 8 ESRC Centre for Society and Mental Health King´s College London London United Kingdom

**Keywords:** COVID-19, mHealth, eHealth, telemedicine, prevention, mental health promotion, intervention, digital mental health, digital intervention, public mental health

## Abstract

**Background:**

Accumulating evidence suggests the COVID-19 pandemic has negative effects on public mental health. Digital interventions that have been developed and evaluated in recent years may be used to mitigate the negative consequences of the pandemic. However, evidence-based recommendations on the use of existing telemedicine and internet-based (eHealth) and app-based mobile health (mHealth) interventions are lacking.

**Objective:**

The aim of this study was to investigate the theoretical and empirical base, user perspective, safety, effectiveness, and cost-effectiveness of digital interventions related to public mental health provision (ie, mental health promotion, prevention, and treatment of mental disorders) that may help to reduce the consequences of the COVID-19 pandemic.

**Methods:**

A rapid meta-review was conducted. The MEDLINE, PsycINFO, and CENTRAL databases were searched on May 11, 2020. Study inclusion criteria were broad and considered systematic reviews and meta-analyses that investigated digital tools for health promotion, prevention, or treatment of mental health conditions and determinants likely affected by the COVID-19 pandemic.

**Results:**

Overall, 815 peer-reviewed systematic reviews and meta-analyses were identified, of which 83 met the inclusion criteria. Our findings suggest that there is good evidence on the usability, safety, acceptance/satisfaction, and effectiveness of eHealth interventions. Evidence on mHealth apps is promising, especially if social components (eg, blended care) and strategies to promote adherence are incorporated. Although most digital interventions focus on the prevention or treatment of mental disorders, there is some evidence on mental health promotion. However, evidence on process quality, cost-effectiveness, and long-term effects is very limited.

**Conclusions:**

There is evidence that digital interventions are particularly suited to mitigating psychosocial consequences at the population level. In times of physical distancing, quarantine, and restrictions on social contacts, decision makers should develop digital strategies for continued mental health care and invest time and efforts in the development and implementation of mental health promotion and prevention programs.

## Introduction

Measures to prevent and control infections during the COVID-19 pandemic such as physical distancing, quarantine, and restrictions on social contacts can have a negative impact on public mental health [1]. This includes an increase in depression, anxiety, loneliness, and perceived stress [2] as well as in risk behaviors such as cannabis and alcohol use [3] in the population. In addition to the immediate effects of the infection control measures, further negative consequences for mental health are to be expected due to the more direct, deleterious effects of COVID-19 (eg, illness anxiety, contamination fears) as well as the economic downturn and recession [4]. Recently reported restrictions in access to, and continuity of, care for individuals with mental disorder caused by infection prevention and control measures in some countries are an additional cause for concern [3,5,6].

Digital interventions that do not require face-to-face contact may play an important role in improving public mental health at times of infection prevention and control measures. They can be broadly grouped as telemedicine and internet-based interventions (hereafter eHealth interventions) [7] and app-based mobile health (mHealth) interventions delivered using smartphones or other mobile devices [8]. These interventions provide a unique opportunity for delivering low-threshold, public mental health care tailored to individual needs and contexts in daily life, outside the clinic [9], even under the restrictive conditions of the COVID-19 pandemic. As smartphones are mostly in close proximity to users, and accessible whenever and wherever it is convenient, the use of mHealth apps in particular represents a powerful approach that allows for the real-time and real-world delivery of intervention components in individuals’ daily lives.

Digital tools may help to mitigate negative psychosocial consequences most effectively if intervention strategies are not only targeted at vulnerable individuals in a clinically high-risk state or with a mental disorder but also at the population level. More specifically, following the seminal “population strategy” advocated by Rose [10], even a small shift in the population’s mean level of mental health, which is continuously distributed in the population, may lead to a substantial reduction of the prevalence of mental health problems. If applied to the current pandemic, a scalable digital public mental health approach may contribute to lower rates of mental disorders by targeting important determinants and shifting the mean level of mental health in the population.

In order to minimize the negative impact of the COVID-19 pandemic on the mental health of the population, digital interventions can be used in the following areas of public mental health provision: primary prevention strategies, including (1) mental health promotion and literacy at the population level; (2) indicated, selective, or universal prevention targeting high-risk individuals, subpopulations, or the entire population, respectively, as well as secondary and tertiary prevention strategies, including (3) treatment and preventive services for people with mental disorders. Indeed, evidence from ad hoc surveys suggests that digital interventions for improving public mental health are urgently needed to address the psychosocial consequences of the COVID-19 pandemic [1-3,11,12]. For example, findings from the serial cross-sectional survey *German COVID-19 Snapshot Monitoring* (COSMO Germany [13]) suggest strong concerns about the economy, social inequalities, and the health care system as well as high levels of psychological distress in the adult general population, particularly among young people [14,15]. Another representative survey (Norstatpanel) found that a staggering 38% of youth met the criteria for moderate or severe mental health problems, even after the most restrictive infection control measures had been lifted [16]. Furthermore, the reported social isolation during the COVID-19 pandemic was associated with levels of psychological distress in a dose-response fashion [16]. Recent evidence also suggests a high subjective demand for digital mental health interventions in the general population and people with a mental disorder [17,18], which is matched with a high and rapidly growing number of mHealth apps available in major app stores, with the strongest growth having been noted for mHealth apps [19]. It has further been reported that the demand for mHealth apps has increased globally by 49% during the COVID-19 pandemic [20], with 73% of psychologically distressed and socially isolated youth in the Norstatpanel survey indicating the use of mHealth apps to be helpful in coping with the ongoing COVID-19 pandemic [16].

Taken together, based on the evidence presented, there is an urgent need for, and high potential in, using digital interventions to improve public mental health and mitigate the negative psychosocial impact of the COVID-19 pandemic. However, evidence-based recommendations for the use of digital interventions during public health crises, including this ongoing pandemic, is currently lacking. The present meta-review aimed to synthesize the available evidence on the theoretical and empirical base of interventions, quality assessments from the user perspective (ie, acceptability, usability, satisfaction), safety, effectiveness, and cost-effectiveness of digital interventions in the area of public mental health provision (ie, mental health promotion and prevention of and treatment for mental disorder).

## Methods

### Overview

A rapid meta-review of systematic reviews on digital public mental health interventions was conducted. For this, PRISMA (Preferred Reporting Items for Systematic Reviews and Meta-Analyses [21]) was used as a guideline for reporting findings. In line with the current state of the art in the development and evaluation of complex digital mental health interventions [8], the following criteria to review the available evidence were used: theoretical and evidence base, quality assessments from the user perspective (ie, acceptability, usability, satisfaction), safety, effectiveness, and cost-effectiveness.

### Search Strategy and Selection Criteria

The MEDLINE, PsycINFO, and CENTRAL databases were searched for systematic reviews and meta-analyses published in the English and German languages from inception to April 2020. An extensive search of bibliographic databases was performed using queries that combined search terms on mental health, public mental health provision, digital eHealth/mHealth interventions (Multimedia Appendix 1), and high-quality reviews (ie, systematic review, meta-analysis) using logical operators. In doing so, database-specific queries were used to ensure semantic equivalence. The queries were launched on May 11, 2020, covering results until April 2020. The results were obtained, and duplicates were removed. References written in English and German were included. No other filters or restrictions were applied.

**Figure 1:**
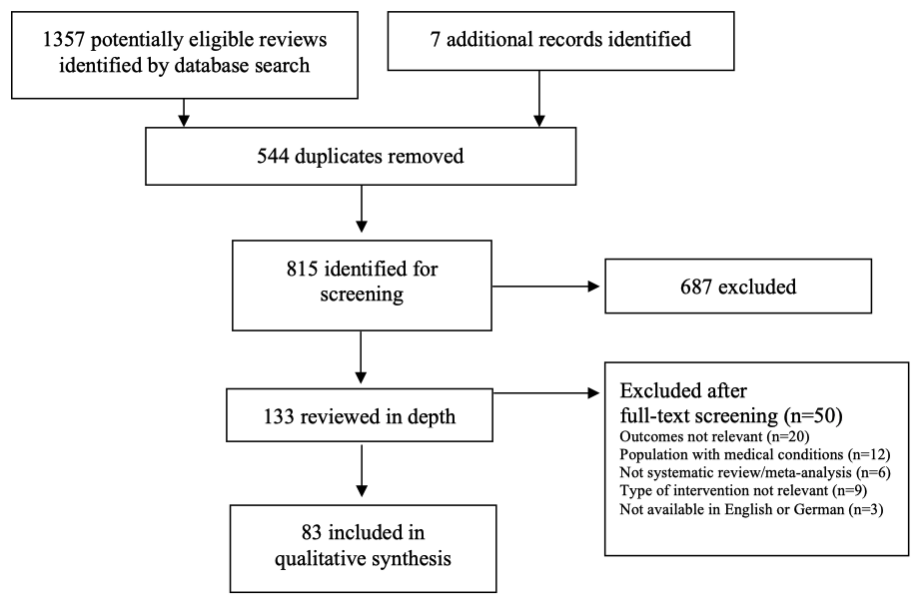
Study selection.

The search criteria were purposefully broad and considered systematic reviews and meta-analyses that investigated digital tools for health promotion, prevention, or treatment of mental health conditions and determinants likely affected by the COVID-19 pandemic (eg, depression, anxiety, psychosis, substance misuse, self-harm, well-being, quality of life, self-esteem, loneliness). Titles and abstracts were screened for inclusion by 1 reviewer (a research assistant). Studies were included if they were published in a peer-reviewed journal, contained original ﬁndings examining the theoretical and evidence base, quality from the user perspective (ie, acceptability, usability, satisfaction), safety, effectiveness, or cost-effectiveness of digital mHealth and eHealth interventions. Due to the rapid meta-review format of our study, conclusions drawn by the authors of the included systematic reviews were reported. The included articles had to be systematic reviews and/or meta-analyses that followed established reporting guidelines (eg, PRISMA [21]). Because of time constraints and the rapid meta-review format of this study, a second reviewer (CR) independently screened a randomly selected subset (40%) of identified studies. The references were categorized as “eligible,” “query,” and “not eligible.” Inclusion and exclusion criteria were applied to references that were queried or eligible. Reviewers were blinded, and potential discrepancies in selection decisions were discussed with another member of the research team. A pilot screening of a randomly selected subset of identified studies (around 5%) was conducted to discuss decisions on categorizing studies at an early stage. As inclusion criteria were purposefully broad, discrepancies between the reviewers (CR and the research assistant) were very low. Full texts of potentially relevant articles were obtained, read, and assessed by 1 reviewer (CR), and data extraction was performed by 3 reviewers (CR and 2 research assistants under the close supervision of CR; see acknowledgments). Reviews and meta-analyses on preprint servers and gray literature were not included. The EndNote reference management software [22] was used to record reviewers’ decisions, including reasons for exclusion. The study selection process was documented using the PRISMA flow diagram (Figure 1).

## Results

### General Findings

The search strategy of our meta-review on digital interventions yielded 815 peer-reviewed systematic reviews and meta-analyses (Figure 1). Of these, 83 references were included in the meta-review. Overall, 44 of the included reviews summarized findings on eHealth interventions and focused on interventions targeting depression (n=19), anxiety (n=22), problematic substance use (n=11), and eating disorders (n=2). Several reviews included interventions that targeted multiple mental health problems. In total, 16 reviews summarized findings on mHealth interventions and focused on depression (n=2), anxiety (n=1), problematic substance use (n=1), and eating disorder (n=1). Similarly, the majority of included reviews targeted various mental health domain. Furthermore, 23 of the included reviews jointly reported the effects of eHealth and mHealth interventions on various mental health outcomes (Multimedia Appendix 2). A complete summary of included reviews on eHealth, mHealth, and mixed interventions are shown in Multimedia Appendix 2, including findings on their theoretical and empirical base, user perspective, safety, effectiveness, and cost-effectiveness.

### Theoretical and Empirical Base

For most eHealth and mHealth interventions in the area of mental health promotion and prevention as well as treatment of mental disorders, the theoretical and empirical base is explicitly mentioned in the description of interventions and are often informed by clinical guidelines and co-designed by service users and mental health professionals [23-26]. This primarily includes evidence-based procedures such as cognitive-behavioral therapy (CBT) or third-wave CBT [23,25,26]. In contrast to digital interventions developed by research groups, prominent mHealth apps available in major app stores do often not provide information on the theoretical or empirical base of their content [8,27-29]. Some mHealth apps may even be harmful and hinder healing processes (eg, asking users to do tasks that are too difficult to complete, presenting means for self-harm as well as lethal means, triggering unwanted distressing memories) [8,27,28].

### Quality Assessments From the User Perspective

Evidence from the included systematic reviews suggests moderate to high levels of acceptance, feasibility, and user satisfaction with eHealth and mHealth interventions for mental health promotion and prevention [30,31] as well as for the treatment of mental health problems [32-41]. This applies, in particular, for interventions including social components [32,42], strategies to promote user adherence [33,43], symptom monitoring [44,45], or a blended-care approach [46].

In terms of safety, data sharing and data safety regulations, as well as aspects of eHealth/mHealth and clinical safety of interventions, were often not explicitly reported or systematically investigated in the identified systematic reviews [47-49] (Multimedia Appendix 2). The descriptions of many eHealth interventions do not make explicit reference to prevailing regulations and clinical guidelines [50]. Furthermore, there is evidence that mHealth apps available in major app stores use problematic data sharing and privacy practices (eg, monetization of sensitive user data through analytics and advertising) [8,27,28].

### Effectiveness of eHealth Interventions

There was good evidence on the effectiveness of telemedical and other eHealth interventions in the field of mental health promotion and prevention, as well as for the treatment of mental health conditions.

#### Mental Health Promotion and Prevention

There have been a number of systematic reviews that aimed to investigate the effectiveness of telemedical and eHealth interventions for mental health promotion and prevention. These interventions have primarily been shown to improve mental health [34], physical activity [34,35], well-being [36,37], stress [23,38], depression [23,36,38,51,52], anxiety [23,36,38,51,52], and alcohol [24,53-56] and cannabis use [57,58] in the general population in addition to dysfunctional cognition and self-esteem in at-risk populations [59,60]. Importantly, effectiveness has been demonstrated across differing age groups, including adults [24,54,59] and adolescents from the general population [34,52,56,61-63], and effect sizes mostly ranged from small to medium. However, evidence on the use of eHealth interventions for the elderly is scarce, although findings from the identified reviews indicated some evidence on the effectiveness of eHealth interventions for reducing social isolation and increasing social participation of people aged 65 years and older [64], which may be of particular interest in the context of the COVID-19 pandemic.

#### Treatment of Mental Health Conditions

There was also strong evidence on the effectiveness of telemedical and eHealth interventions in the provision of treatment and services for people with mental disorder. This included anxiety disorders [65-68], depression [60,61,65-67,69-73], substance abuse [54,74-76], eating disorders [77], and severe mental illness [78], with overall small to medium effect sizes, not only with regard to the reduction of relevant symptoms but also improvements in dysfunctional cognition [60], self-esteem [60], and quality of life [66]. Some of the identified studies have even reported medium to large effect sizes for cognitive-behavioral eHealth interventions that aimed to reduce symptoms of depression [79].

The effectiveness of telemedicine interventions that use videoconference tools or the telephone has also been well documented in depressive [80-83], anxiety [80,83-85], and psychotic disorders [86], with comparable effects for online group and individual therapy sessions [87,88], compared with conventional (offline) therapy sessions. Superior effectiveness was observed for interventions adopting a blended-care approach combining eHealth with conventional intervention components [46,54,71].

Overall, findings suggest that the evidence on long-term effects and noninferiority compared to conventional therapy and active control conditions remains limited [79,81,82,86,87]. There is also limited evidence on the impact of telemedical and eHealth interventions on underlying processes and mechanisms of change [89].

### Effectiveness of mHealth Interventions

While there is some initial evidence on the effectiveness of mHealth interventions to improve physical activity [90-95], stress appraisal [96,97], depression [26,96-100], anxiety [25,26,96,97], and alcohol and substance use [55,96,98,101-103], with small to medium effect sizes in all areas of public mental health provision, the amount of research to investigate this issue remains, overall, limited [104-108]. Only a minority of mHealth interventions were found to use more advanced techniques (accelerometer, GPS) to inform the delivery of intervention components [25,89,92]. In addition, a substantial difference was found between mHealth apps available in major app stores, for which there is no or only very limited evidence on their effectiveness [29,108-111], and mHealth interventions developed by research groups. Similar to eHealth interventions, evidence on long-term effects and on underlying processes and mechanisms of action remains very limited.

### Cost-effectiveness

There is some evidence on the cost-effectiveness of eHealth interventions for depression and anxiety in primary care settings when compared to care as usual and waiting list control conditions [51] as well as for a range of mental disorders when compared to conventional CBT [112,113]. However, as only a few systematic reviews have systematically investigated the cost-effectiveness of digital interventions to date, these findings should be interpreted with caution. While there is some evidence on the cost-effectiveness of mHealth interventions (eg, for digital monitoring and feedback in depression) from individual studies [18], evidence summarized at the level of systematic reviews is very limited.

## Discussion

### Principal Results

Evidence-based eHealth and mHealth interventions may play a central role in areas of public mental health provision (ie, mental health promotion, as well as prevention of and treatment for mental disorders) to mitigate the negative consequences of the COVID-19 pandemic. To date, however, evidence-based recommendations on existing digital interventions that have been developed and evaluated in recent years are lacking. This meta-review was the first to review the available evidence on the theoretical and empirical base, quality assessments from the user perspective (ie, acceptability, usability, satisfaction), safety, effectiveness, and cost-effectiveness of digital interventions in the area of public mental health provision, that is, mental health promotion at the population level, indicated, selective, or universal prevention targeting high-risk individuals, subpopulations, or the entire population as well as treatment and services for people with mental disorders.

First, there was robust evidence on the effectiveness of telemedical eHealth interventions and initial evidence on the effectiveness of mHealth interventions in relation to mental health outcomes likely affected by the COVID-19 pandemic (eg, anxiety, depression), especially if interventions are informed by clinical guidelines and co-designed by service users and mental health professionals. Second, effectiveness, acceptability, feasibility, and user satisfaction have been described to be particularly high if digital interventions are embedded in a therapeutic context and include some form of social interaction with a mental health professional (blended-care approach). Third, some of the included systematic reviews and meta-analyses suggest noninferiority of effectiveness for some eHealth interventions as compared to traditional face-to-face therapy, but further replication is needed before firm conclusions can be drawn. Thus, in order to exclude the risk of infection in the current public health crisis, clinicians and other health professionals may consider combining differing types of digital interventions (eg, counseling or psychotherapy using videoconference software augmented by a smartphone-based mHealth app) as this approach may be particularly promising given the current evidence base and reflects a novel digital version of the blended-care approach. However, more research is needed to investigate long-term treatment effects and effects of symptom monitoring on mental health outcomes. Notably, the evidence on the use of digital interventions for the elderly and children is very limited. This is an important finding as these age groups may be particularly challenged by the current pandemic. Fourth, most studies to date do not specifically investigate the additive effects on health-related outcomes when using more advanced techniques (eg, accelerometer, GPS) to further personalize the delivery of intervention components, gamification elements, and the integration of other technologies such as wearables, although it has been described to be potentially beneficial in some of the included reviews [25,89,92,114]. Fifth, the theoretical basis of most digital interventions that have been described in previous reviews were found to be CBTs or third-wave CBTs as they may be particularly amendable to translation into digital intervention components [23,25,26]. Thus, clinicians with an expertise in CBT techniques may find it easier to purposefully incorporate intervention components delivered using digital tools in their daily clinical routines. However, findings suggest that there is a need to further improve the theoretical foundation of digital intervention, particularly mHealth interventions publicly available in major app stores. Sixth, the data available on the process quality and cost-effectiveness of eHealth and mHealth interventions are limited. Seventh, users frequently report concerns about data safety and privacy [115]. While eHealth and mHealth interventions developed and evaluated by research groups generally comply with the General Data Protection Regulation (in European countries) and work in accordance with Good Clinical Practice standards, the contents of many mHealth apps currently available in major app stores do not explicitly refer to existing clinical guidelines and recommendations by learned societies [50,116]. There are a number of reviews that have concluded that mHealth apps have problematic data-sharing and privacy practices [8,27,28] and that there may not only be a lack of quality of offered content but even harmful intervention components. In addition, although not specifically reported in included systematic reviews and meta-analyses, the recent surge in the use of popular and freely available platforms (eg, Zoom, Skype) rather than secured platforms to provide online mental health services may be another cause of concern [117] as these platforms mostly do not comply with national standards for sensitive patient data protection. In order to demonstrate user safety, clinical guidelines should be explicitly taken into account and advice by mental health professionals, learned societies, and IT (information technology) professionals actively incorporated. Overall, apps available in app stores should be used with caution due to risks in data and clinical safety as well as a lack of evidence on their effectiveness.

### Limitations

This meta-review has several limitations. Because of time constraints and the rapid meta-review format of this study, the quality of included systematic reviews was not evaluated using established assessment tools (eg, the AMSTAR 2 [A Measurement Tool to Assess Systematic Reviews] checklist [118]). Along similar lines, the conclusions drawn in this meta-review on the quality of evidence are largely based on quality assessments undertaken in the included systematic reviews and meta-analyses. However, if the quality of evidence was not systematically evaluated using a standardized approach, it is indicated in Multimedia Appendix 2. Additionally, only 1 reviewer screened identified articles while a second reviewer independently screened a randomly selected subset (40%) of studies. However, this meta-review was conducted in line with the state of the art of conducting rapid reviews [119]. Furthermore, the World Health Organization has explicitly recommended rapid reviews for evidence synthesis during the ongoing public health crisis, given these are urgently needed for policy makers and the public [120].

In considering the urgent need of continued access to mental health care for vulnerable individuals during the COVID-19 pandemic, and the importance of developing and implementing public mental health prevention and promotion strategies, digital interventions should be provided by public health services and routinely offered when infection control measures are implemented during pandemics. Since there is currently no direct evidence on digital interventions that aim to minimize the psychosocial impact of previous coronavirus and influenza virus outbreaks, digital interventions should be developed and evaluated by research groups in close collaboration with relevant stakeholders to ensure established standards for investigating quality from the user perspective, effectiveness, and cost-effectiveness are met. Importantly, evidence-based digital interventions are scalable and can be rapidly delivered at the population level. This may facilitate delivering personalized care and minimizing the negative impact of the COVID-19 pandemic on public mental health.

### Conclusions

Decision makers and stakeholders, including policy makers, technology companies, and public health professionals, should join forces to develop evidence-based strategies for mental health care in the area of public mental health provision, especially in moments of public health crises. As studies from previous pandemics, as well as accumulating evidence from the COVID-19 pandemic, suggest a negative impact on public mental health, the development and implementation of mental health promotion and prevention strategies at the population level may be an important measure to improve public mental health. Digital interventions that incorporate contact with mental health staff in a blended-care approach may be particularly suited to alleviate mental health burden in help-seeking individuals. At times of COVID-19 and physical distancing measures, this may be translated into a digital blended-care approach by combining telemedical with internet-based eHealth or smartphone-based mHealth interventions. Furthermore, efforts should be made to systematically evaluate currently available digital interventions based on established criteria of digital mental health and mental health services research, as demonstrated by recent initiatives (eg, National Health Service Apps Library in the United Kingdom; Platform for Digital Health Applications in Germany; App Evaluation Database provided by the Division of Digital Psychiatry, Beth Israel Deaconess Medical Center, in the United States) [121-123]. This would systematize the search for evidence-based mHealth apps and thus allow clinicians and interested users to make more informed decisions on the quality of currently available digital interventions. There is also a need to carefully examine the role of social inequalities and the related digital divide as well as possible barriers (eg, disproportional access to necessary technologies, educational requirements, language skills, cultural factors, motor or cognitive impairments), which can influence the access to and use of the information platforms of digital mental health interventions.

## Appendix

Multimedia Appendix 1 The full electronic search strategy for one database.

Multimedia Appendix 2 Complete summary of included reviews on eHealth, mHealth, and mixed interventions. Findings on target populations, intervention components, theoretical and evidence base on process/outcomes, primary outcomes and quality of evidence, secondary outcomes, quality from the user perspective, safety, and cost-effectiveness are shown.

## Abbreviations

AMSTAR 2: A Measurement Tool to Assess Systematic Reviews
CBT: cognitive-behavioral therapy
COSMO Germany: German COVID-19 Snapshot Monitoring
IT: information technology
mHealth: mobile health
PRISMA: Preferred Reporting Items for Systematic Reviews and Meta-Analyses
