# Supplementary material for: Evidence Synthesis of Digital Interventions to Mitigate the Negative Impact of the COVID-19 Pandemic on Public Mental Health: Rapid Meta-review
Source: J Med Internet Res. 2021 Mar 10;23(3):e23365. doi: 10.2196/23365 (PMC7951054; doi:10.2196/23365)
Supplement: Multimedia Appendix 1 [file jmir_v23i3e23365_app1.docx]

**Multimedia Appendix 1**

This is a Multimedia Appendix to a full manuscript published in the J Med Internet Res. For full copyright and citation information see http://dx.doi.org/10.2196/23365.

Search strategy

Database: Ovid MEDLINE(R) <1946 to April Week 4 2020>

Search Strategy:

--------------------------------------------------------------------------------

1 exp Mental Health/ (37303)

2 exp Psychopathology/ (7194)

3 exp Mental Disorders/ (1225111)

4 "mental health".tw. (113359)

5 "mental dis*".tw. (37183)

6 psychopatholog*.tw. (35952)

7 "mental ill*".tw. (25880)

8 "psychiatric dis*".tw. (41201)

9 SMD.tw. (6004)

10 exp Depression/ (116762)

11 exp Depression, Postpartum/ (5418)

12 exp Depressive Disorder, Major/ (29572)

13 exp Depressive Disorder/ (107891)

14 exp Dysthymic Disorder/ (1135)

15 exp Mood Disorders/ (120656)

16 exp Affect/ (32941)

17 exp Emotions/ (237356)

18 exp Emotional Regulation/ (195)

19 depress*.tw. (394544)

20 dysthymi*.tw. (2850)

21 melanchol*.tw. (2736)

22 mood*.tw. (63196)

23 affect*.tw. (1563493)

24 emotion*.tw. (160488)

25 sad.tw. (8270)

26 lonel*.tw. (5604)

27 exp Anxiety/ (83635)

28 exp Anxiety Disorders/ (78672)

29 exp Phobic Disorders/ (11222)

30 exp Agoraphobia/ (2581)

31 exp Performance Anxiety/ (140)

32 exp Panic Disorder/ (6843)

33 exp Panic/ (2598)

34 exp Fear/ (32507)

35 anxi*.tw. (172074)

36 agoraphobi*.tw. (3157)

37 GAD.tw. (7875)

38 panic*.tw. (16859)

39 fear*.tw. (68150)

40 phobi*.tw. (10167)

41 fright*.tw. (2288)

42 exp Obsessive Behavior/ (1404)

43 exp Obsessive-Compulsive Disorder/ (14429)

44 OCD.tw. (7833)

45 obsessive*.tw. (15408)

46 exp Psychotic Disorders/ (51615)

47 exp Paranoid Disorders/ (4078)

48 exp Schizophrenic Psychology/ (33207)

49 exp Schizophrenia/ (103737)

50 exp Schizophrenia, Disorganized/ (538)

51 exp Schizophrenia, Paranoid/ (4110)

52 exp Schizophrenia, Catatonic/ (576)

53 exp Prodromal Symptoms/ (1578)

54 exp Schizophrenia, Childhood/ (1549)

55 psycho*.tw. (537844)

56 schizo*.tw. (124146)

57 paranoi*.tw. (7341)

58 hallucinat*.tw. (12064)

59 delusion*.tw. (9419)

60 exp Bipolar Disorder/ (39900)

61 bipolar*.tw. (53604)

62 mani*.tw. (498873)

63 hypomani*.tw. (2534)

64 exp Personality Disorders/ (41057)

65 exp Borderline Personality Disorder/ (6757)

66 exp Antisocial Personality Disorder/ (9455)

67 exp Dependent Personality Disorder/ (226)

68 exp Schizoid Personality Disorder/ (593)

69 exp Histrionic Personality Disorder/ (3993)

70 "personality disorder*".tw. (17440)

71 histrionic.tw. (598)

72 borderline.tw. (37356)

73 dependen*.tw. (1444582)

74 exp Narcissism/ (2862)

75 narci*.tw. (3641)

76 exp Attention Deficit Disorder with Hyperactivity/ (28293)

77 exp "Attention Deficit and Disruptive Behavior Disorders"/ (32289)

78 exp Attention/ (77279)

79 ADHD.tw. (20249)

80 attention.tw. (325691)

81 exp Child Behavior Disorders/ (20188)

82 exp Conduct Disorders/ (3363)

83 "callous unemotional".tw. (597)

84 "dysfunction of behavio*".tw. (275)

85 "social behavio*".tw. (11306)

86 antisocial.tw. (7625)

87 "anti-social".tw. (354)

88 dyssocial.tw. (34)

89 dissocial.tw. (175)

90 "aggressive behavio*".tw. (13076)

91 defiant.tw. (2087)

92 delinquen*.tw. (6130)

93 "conduct disorder*".tw. (4095)

94 exp Autistic Disorder/ (20200)

95 autis*.tw. (37691)

96 exp Adjustment Disorders/ (4204)

97 exp Social Adjustment/ (23204)

98 adjustment.tw. (139354)

99 exp Cognition Disorders/ (92327)

100 exp Intellectual Disability/ (95241)

101 cognition*.tw. (57240)

102 intellect*.tw. (34165)

103 disabilit*.tw. (153891)

104 learning.tw. (225885)

105 exp "Sleep Initiation and Maintenance Disorders"/ (13034)

106 exp Sleep Disorders, Circadian Rhythm/ (2269)

107 exp Sleep/ (78550)

108 sleep.tw. (138836)

109 insomnia.tw. (16769)

110 nightmares.tw. (1547)

111 exp Stress Disorders, Traumatic/ (35714)

112 exp Stress Disorders, Post-Traumatic/ (32062)

113 exp Stress, Psychological/ (129346)

114 exp Stress, Physiological/ (214074)

115 stress.tw. (611314)

116 distress*.tw. (103640)

117 "post-trauma*".tw. (26218)

118 PTSD.tw. (19272)

119 exp Substance-Related Disorders/ (275494)

120 "substance disorder*".tw. (310)

121 (substance adj3 abus*).tw. (23317)

122 addict*.tw. (53307)

123 cannabis.tw. (13262)

124 tobacco.tw. (84037)

125 exp Alcoholism/ (74611)

126 exp Alcohol Drinking/ (68175)

127 alcohol*.tw. (279470)

128 amphetamine.tw. (20830)

129 hallucinogens.tw. (1264)

130 exp "Feeding and Eating Disorders"/ (30305)

131 exp Anorexia Nervosa/ (12849)

132 exp Binge-Eating Disorder/ (1420)

133 exp Bulimia Nervosa/ (2321)

134 exp Eating/ (71950)

135 exp Bulimia/ (5444)

136 exp Anorexia/ (4933)

137 "eating disorder*".tw. (16385)

138 "body-imag*".tw. (10541)

139 "binge-eating".tw. (4751)

140 bulimi*.tw. (7600)

141 anorexi*.tw. (29319)

142 exp Sexual Dysfunctions, Psychological/ (26480)

143 sexual.tw. (166394)

144 orgasm.tw. (2590)

145 desire.tw. (27818)

146 erectile.tw. (18047)

147 ejaculation.tw. (6367)

148 dyspareunia.tw. (3342)

149 exp Self-Injurious Behavior/ (69868)

150 "self-injur*".tw. (3839)

151 "self-harm".tw. (4156)

152 suicid*.tw. (66400)

153 "at risk*".tw. (147796)

154 "high risk*".tw. (236307)

155 ARMS.tw. (49756)

156 UHR.tw. (712)

157 vulnerab*.tw. (107836)

158 exp Anhedonia/ (959)

159 anhedon*.tw. (3456)

160 exp Motivation/ (167764)

161 exp Reward/ (21181)

162 motivation.tw. (49856)

163 exp Developmental Disabilities/ (19858)

164 exp Personality Development/ (153603)

165 exp Adaptation, Psychological/ (125798)

166 development*.tw. (2017494)

167 internali*.tw. (54249)

168 externali*.tw. (12204)

169 "quality of life".tw. (225450)

170 happiness.tw. (5276)

171 satisfact*.tw. (227385)

172 "social support".tw. (31355)

173 pleasure.tw. (5782)

174 gratitude.tw. (1050)

175 compassion*.tw. (7850)

176 well-being.tw. (60119)

177 resilien*.tw. (22733)

178 or/1-177 (8415847)

179 exp Telemedicine/ (27662)

180 exp Internet-Based Intervention/ (89)

181 internet.tw. (39914)

182 ehealth.tw. (1775)

183 "web-based".tw. (23543)

184 "e-health".tw. (1709)

185 telemed*.tw. (8716)

186 telehealth*.tw. (2984)

187 teletherap*.tw. (1266)

188 uhealth.tw. (1)

189 "u-health".tw. (19)

190 eTherap*.tw. (9)

191 "e-Therap*".tw. (373)

192 exp mobile application/ (5560)

193 exp Smartphone/ (4124)

194 "mobile health".tw. (1928)

195 mHealth.tw. (1600)

196 m-health.tw. (279)

197 app.tw. (19176)

198 "app-based".tw. (242)

199 "mobile app*".tw. (2150)

200 "mobile-based".tw. (245)

201 "phone-based".tw. (773)

202 smartphone.tw. (5176)

203 "smartphone-based".tw. (910)

204 "digital tool".tw. (45)

205 "digital assist*".tw. (965)

206 apps.tw. (3316)

207 or/179-206 (115779)

208 exp Primary Prevention/ (150004)

209 exp Secondary Prevention/ (20087)

210 exp Health Promotion/ (75852)

211 exp Early Intervention, Educational/ (2987)

212 exp Internet-Based Intervention/ (89)

213 exp Early Medical Intervention/ (2920)

214 prevention.tw. (462307)

215 intervention*.tw. (812225)

216 "health promotion*".tw. (25265)

217 treatment*.tw. (3843362)

218 "health service".tw. (39081)

219 therap*.tw. (2375507)

220 counselling.tw. (22841)

221 counseling.tw. (56370)

222 or/208-221 (6099759)

223 "systematic review".ti. (90092)

224 178 and 207 and 222 and 223 (825)

***************************
