# Supplementary material for: Evidence Synthesis of Digital Interventions to Mitigate the Negative Impact of the COVID-19 Pandemic on Public Mental Health: Rapid Meta-review
Source: J Med Internet Res. 2021 Mar 10;23(3):e23365. doi: 10.2196/23365 (PMC7951054; doi:10.2196/23365)
Supplement: Multimedia Appendix 2 [file jmir_v23i3e23365_app2.docx]

**Multimedia Appendix 2**

This is a Multimedia Appendix to a full manuscript published in the J Med Internet Res. For full copyright and citation information see http://dx.doi.org/10.2196/23365.

Complete summary of included reviews on eHealth, mHealth, and mixed interventions. Here, findings on secondary outcomes, quality from the user perspective, safety, and cost effectiveness are shown which have been omitted in the manuscript.

**Table S1.** Included reviews on eHealth interventions.

| Reference | Meta-analyses | Population | Intervention components | Theoretical and evidence base on process/outcomes | Primary outcomes and quality of evidence | Secondary outcomes | Quality from user perspective | Safety | Cost effectiveness |  |
| --- | --- | --- | --- | --- | --- | --- | --- | --- | --- | --- |
| Mental Health Promotion and Prevention | | | | | |  |  |  |  |  |
| Alkhaldi et al. (2016) ^33^ | Yes | Adult general population | Technology-based engagement promotion strategies (eg, text messages, self-monitoring, F, support) | Not reported | Small to moderate positive effects on technology-based strategies for promoting engagement with digital interventions as compared to no strategy; quality of evidence: moderate (assessed using Cochrane risk-of-bias tool) | Not reported | Possible increase of engagement with technology-based strategies | No adverse events reported | Not reported |  |
| Elaheebocus et al. (2018) ^32^ | No | Adult general population | Identity representation, communication, peer grouping, data sharing, competition/gamification, activity data viewing, online social network | Social Behavior Change Technique | Positive effects on outcomes (eg, increased PA, lower alcohol intake) in most behavioral interventions that included social media features, 28% no effect; quality of evidence: low (eg, small samples, heterogeneous interventions); no standardized approach was used to assess the quality of included studies | Social media features might be relevant for improving variety of health outcomes | Lowered helpfulness, increased satisfaction, and motivation reported | No adverse events reported | Not reported |  |
| Cotie et al. (2018) ^35^ | Yes | Adult general population | Multimodal/unimodal websites: information, PA tracking tools, online discussion forums; prompts (eg, reminders to exercise, track movement) | Social Cognitive Theory, Transtheoretical Behavioral Theory | Moderate improvement in PA; no improvement in obesity-related outcomes; quality of evidence: moderate (assessed using the GRADE approach) | eHealth intervention as a tool for promotion of lifestyle improvements highly accessible, time efficient | Not reported | Not reported | Cost effectiveness reported; no data on savings provided |  |
| Deady et al. (2017) ^59^ | Yes | Adult general population | Behavioral and problem-solving therapy, mindfulness, situational analysis, skills program, relaxation self-help program, persuasive framing, PE | CBT, third wave CBT, ACT, PE | Small positive effects on depression and anxiety; similar effect sizes for universal and indicated/selective interventions; quality of evidence: moderate (assessed using the Downs and Black checklist) | Inadequate evidence on medium to long-term effect and reduction of incidence of mental disorders | Not reported | Not reported | Not reported |  |
| Heber et al. (2017) ^23^ | Yes | Adult general population | Mindfulness, stress and mood management, problem solving, ER, coping with stress, ACT, skills, relaxation | CBT, third wave CBT, ALT | Small effect on stress, depression and anxiety; guided interventions more effective than unguided interventions; small effects for short interventions (≤4 weeks), moderate effects for medium-long interventions (5-8 weeks; small-moderate effect for CBT and third wave CBT; small effect for alternative interventions; small-moderate effect up to 6 months follow-up for computer-based stress-management interventions; quality of evidence: low (assessed using the Cochrane risk-of-bias tool) | Not reported | Not reported | Not reported | Cost effectiveness reported; no data on savings provided |  |
| Ennis et al. (2018) ^31^ | No | Trauma-exposed individuals | PE, interactive games, cognitive and behavioral principles, peer support, MI | CBT | No effects on various symptom domains for selected interventions, significant effects for indicated interventions as compared to active control conditions; quality of evidence: fair to good (assessed using the Downs and Black checklist) | No long-term follow-up data, heterogeneity of studies and outcomes | Not reported | Adverse events reported in one study | Potential low cost; no data on savings provided |  |
| Pennant et al. (2015) ^52^ | Yes | Young individuals (aged 5-25 years) with risk of developing anxiety disorder or depression; young individuals from the general population | Supportive text, animation, photographs, videos, rewards, games, homework, web pages with reading, quizzes, F, phone calls, interactive fantasy adventure game, depression monitor, diary, counter-thought generator | CBT | Medium effects on anxiety and depression in populations at risk; small effects on anxiety and depression in general population; inconsistent findings in children; quality of evidence: low (assessed using the GRADE approach) | Sparse evidence for other computerized interventions (computerized but not cCBT) | Not reported | Not reported | Not reported |  |
| Fleming et al. (2014) ^61^ | No | Young individuals (aged 9-25 years) with depressive symptoms | Serious games: supported and fully self-help interventions; F, conflict/competition, interaction, PE | CBT | Small treatment effects on depressive symptoms; quality of evidence: low (eg, small samples, heterogeneous interventions); no standardized approach was used to assess the quality of included studies | Not reported | Promising findings on adherence, but moderate satisfaction | Not reported | Not reported |  |
| Flujas-Contreras et al. (2019) ^37^ | Yes | Parents of children with mental or physical health problems | PE, self-care, positive parenting, coping strategies, children´s contingency management, problem solving games | CBT, Triple P | Moderate effect sizes on various mental health domains; small effect size on parental self-efficacy, no effects on parental stress; Triple P: small effect size; quality of evidence: high (assessed using the Cochrane risk-of-bias tool and the Moncrieff Scale) | Positive effects on parenting and emotional wellbeing of parents and Children; increased attendance and participation level in technology-based treatment (compared with traditional interventions) | Not reported | Not reported | Not reported |  |
| Boumparis et al. (2019) ^57^ | Yes | School and high school students (aged 12-20); problematic cannabis users (aged 16-40) | Parent-involvement-program, climate school course, MI, brief interventions, solution focused approaches, skills-based prevention programs, community reinforcement approach | CBT, Person-centered Therapy, Social Influence Theory | Small effects on cannabis use in students and problematic cannabis users as compared to controls; effects maintained in students at 12-month follow-up but not in problematic cannabis users; quality of evidence: low (assessed using the Cochrane risk-of-bias tool) | Not reported | Not reported | Not reported | eHealth  intervention significantly more cost-effective than  therapist-delivered intervention; no data on savings reported |  |
| Hadjistavropoulos et al. (2020) ^54^ | Yes | Adults general population; service users with alcohol use disorder recruited from specialized mental health services | Information on alcohol, preparing for change exercises, skills training, drinking diary, online discussion forum, automated motivational text or email prompts, blood-alcohol-concentration calculator | CBT | Self-guided iCBT significantly more effective in reducing alcohol consumption when compared to information about alcohol and waitlist control conditions; therapist-guided iCBT more effective as compared to waitlist (medium-large effect) control condition; quality of evidence: moderate (assessed using the Cochrane risk-of-bias tool) | Guided ICBT can be useful for patients at risk for treatment failure | Not reported | Not reported | Not reported |  |
| Tait et al. (2010)^56^ | No | Young people with substance misuse | Interactive assignments, video clips, personalized normative F, information, interactive online alcohol education, assessment | Not reported | Small effect in preventing development of alcohol-related problems among people who were nondrinkers at baseline; effects similar to brief in-person interventions; quality of evidence: low (eg, small sample sizes, mostly short-term effects investigated, considerable heterogeneity), no standardized approach was used to assess the quality of included studies | Insufficient data to assess utility of SC interventions | Not reported | Not reported | Not reported |  |
| Treatment |  |  |  |  |  |  | | | |  |
| Arguel et al. (2018) ^50^ | Yes | Adults with various mental disorders | Social network interventions (eg, social networks and forums, discussion groups) | Social Cognitive Theory; Theory of Planned Behavior, Technology Acceptance Model. Perceived Risk Influence | Most common theory for online social network interventions is Social Cognitive Theory, quality of evidence: low (assessed using the Cochrane risk-of-bias tool) | Lack of guidelines for online social network interventions; no reference to health psychology theories | Not reported | Not reported | Not reported |  |
| Lin et al. (2019) ^75^ | No | General population, patients with substance use disorder, primary care patients | Psychotherapy: SC, MI, individual counselling sessions, group therapy focused on relapse prevention, ME, combination of videoconference & methadone treatment, PE | CBT, CBI | Superior treatment retention for telemedicine in one study; lower dropout rate in one study; quality of evidence: moderate (eg, retrospective studies, moderate risk of bias) (assessed using the Cochrane risk-of-bias tool) | Telemedicine interventions are promising; especially useful when other treatments are less available; high satisfaction; substantial methodological limitations | High satisfaction; comparable to in-person treatment; technical challenges | Not reported | Higher costs for videoconference than treatment via phone; no data on savings provided |  |
| Simon et al. (2019) ^39^ | No | Adults with PTSD | PE; stress management techniques; cognitive restructuring/trauma processing; relapse prevention. | CBT | High levels of acceptability; quality of evidence: moderate (assessed using the Cochrane risk-of-bias tool) | Greater dropout from ICBT than waitlist; no difference in dropout between ICBT and I-non-CBT | High satisfaction in experimental treatment groups; moderate to high acceptability | Two participants in intervention condition reported clinically significant increase in depression, one in anxiety symptoms (both experienced death of family member during treatment) | ICBT for individuals with PTSD offers potential  as a cost-effective, timely and accessible treatment  choice; no data on savings provided |  |
| Richards et al. (2012) ^70^ | Yes | Adults with symptoms of depression | CBT-ID, Beating the Blues (BTB), MoodGym, Sadness Program, Overcoming Depression on the Internet (ODIN), Color your Life 5 | CBT | Overall medium effect size; large effect and greater retention for therapist-supported studies; small effect for studies without support; quality of evidence: high, but high risk of missing data (assessed using the Cochrane risk-of-bias tool) | Similar effect for administrative-supported studies; support only significantly different between studies with no support vs therapist support | 70-80% reported general satisfaction, perceived benefit, program equaled or was better than a usual therapy; 95% recommended intervention | Not reported | Unsupported programs have the potential to increase access, at minimal cost, especially where human resources are limited; no data on savings provided |  |
| Sierra et al. (2018) ^79^ | Yes | Individuals with depression | Behavioral activation, acceptance and commitment therapy | third wave CBT | Medium to large effect sizes comparing online intervention vs. waitlist or TAU; quality of evidence: low (eg, high attrition to post-measure in some studies, high variability in sample size), no standardized approach was used to assess the quality of included studies | Not reported | Not reported | Not reported | Cost effectiveness reported; no data on savings provided |  |
| Erbe et al. (2017) ^46^ | No | Adults with depression, anxiety, and/or substance abuse | Blended Interventions: treatment programs that use elements of both face-to-face and internet-based interventions; web-based programs with modules such as cognitive, behavioral and emotion-focused interventions, email support, PE, group chats | CBT | Blended interventions are feasible and more effective than waiting list control conditions; quality of evidence: low; no standardized approach was used to assess the quality of included studies | Blended therapy time effective; lower dropout rates and/or greater abstinence rates of patients with substance abuse, may help maintain effects of inpatient therapy, may increase effects of psychotherapy | Blended Interventions can lead to lower dropout rates and greater abstinence rates of patients with substance abuse | Not reported | Cost effectiveness reported; no data on savings provided |  |
| Lau et al. (2016) ^62^ | Yes | Population (aged 7-80) with symptoms of depression, PTSD, autism, ADHD, alcohol use disorder | Goal-oriented, cognitive training games (physical, emotional, cognition, skills), PE | Goal-oriented, Problem-Solving, Cognition Training, and Games | Moderate effect on various symptom domains; quality of evidence: moderate, risk of bias unclear (incomplete reporting) (assessed using the Cochrane risk-of-bias tool) | Not reported | Not reported | Not reported | Not reported |  |
| Irvine et al. (2020) ^88^ | Yes | Heterogenous sample (eg, students, adults with and without diagnoses of mental disorders, adults referred to mental health services) | Counselling, CBT techniques used by therapist, solution focused therapy, peer counselling, Employee Assistant Program | CBT | Telephone sessions shorter than face-to-face sessions; no significant difference in therapeutic alliance, disclosure, empathy, attentiveness; more active participation in telephone mode; quality of evidence: low (eg, small sample sizes, observational studies, high levels of inconsistency), no standardized approach was used to assess the quality of included studies | Not reported | No significant differences in alliance | Going out to engage in a face-to-face appointment may be essential for therapeutic process | Cost effectiveness reported; no data on savings provided |  |
|  |  |  |  |  |  |  |  |  |  |  |
| Gentry et al. (2019) ^87^ | No | Veterans with PTSD, opioid-dependents; women with HIV; cancer patients, inmates in seclusion, smokers, diabetes patients | Group therapy via VTC services eg, relapse control therapy, coping skills, healthy relationship educational program, cognitive processing therapy, relaxation response resilience program, mindfulness, SC, self-management education, chat group, video group, psychosocial support | CBT, ACT, PE | No differences between VTC and face-to-face group sessions on treatment outcomes (eg, PTSD symptoms); effects of VTC comparable to face-to-face treatment; mild decreases of therapeutic alliance in VTC; quality of evidence: moderate (assessed using the quality of evidence criteria of the US Preventive Services Task Force) | VTC is feasible | High satisfaction, generally positive F from participants (despite technical challenges) | Not reported | Not reported |  |
| Grist et al. (2013) ^65^ | Yes | Participants with common mental disorders | eg, Beating the Blues, MoodGym, Panic Online, Color Your Life, DE-STRESS, FearFighter, Coping With Depression; support via email, phone, web forum, text messages | CBT | Medium effect size of iCBT on various mental health outcomes, comparable to face to face CBT; iCBT significantly more effective than waitlist and active control; quality of evidence: moderate (assessed using the Cochrane risk-of-bias tool) | Moderators: mean age of study sample (negative correlation of age and effectiveness), type of control group | Not reported | Not reported | Cost effectiveness reported; no data on savings provided |  |
| Axelsson et al., (2019) ^112^ | Yes | Adult patients with health anxiety | Cognitive restructuring, exposure-based techniques | CBT | Moderate to large effects of iCBT on health anxiety (superior to active controls in two studies), small effect on QoL; comparable effects of iCBT to face-to-face-CBT; quality of evidence: moderate (eg, small effect of publication bias, substantial heterogeneity), low risk of bias (assessed using the Cochrane risk-of-bias tool) | 2/3 responded to ICBT, 1/2 achieve remission; control condition as moderator of effect size; effects in routine care, effects mostly sustained at 12-month follow-up | Not reported | Not reported | Therapist-guided ICBT cost-effective  when compared to passive controls  and internet-delivered behavioral stress management; no data on savings provided |  |
| Berryhill et al. (2019) ^80^ | No | Adult patients with anxiety disorder | Video-conferencing psychological therapy | CBT, ACT, Metacognitive Therapy | Small to large improvements on anxiety; quality of evidence: moderate (assessed using the Effective Public Health Practice Project quality assessment tool) | No differences between videoconferencing and face-to-face groups | Not reported | Not reported | Not reported |  |
| Bolton et al. (2015) ^84^ | Yes | Adults with traumatic experience | Hybrid design: internet and real-time (telephone calls, initial face-to-face introductory meeting) delivery of intervention components or asynchronous (email) communication | CBT | Medium-large improvements in cognitive and behavioral symptoms of depression, generalized anxiety and posttraumatic stress; quality of evidence: moderate (eg, underpowered, rare blinded group allocation) (assessed using the Quality Index) | No superiority of telepsychology to face-to-face psychotherapy in long-term maintenance, eHealth as potential short-term treatment option; contributes to immediate, positive changes in 1/3 physical outcomes and 10/20 psychosocial outcomes | High consumer satisfaction and treatment acceptance, participation rate (83%) comparable to face-to-face psychotherapy, participants favored short-term treatment conditions | Not reported | Not reported |  |
| Castro et al. (2020) ^81^ | Yes | Depressed adults with no comorbid somatic disorders | Workbook including exercises, skills trainings and coping strategies, information about local mental health services, pedometer | CBT | Large reduction in depressive symptoms compared to control; nonsignificant small effect of telephone-administered psychotherapy compared to active comparators; quality of evidence: low (assessed using the Cochrane risk-of-bias tool) | Not reported | Mean adherence: 73% | Not reported | Not reported |  |
| Stech et al. (2020) ^68^ | Yes | Adults with an at-risk mental state or a diagnosis of panic disorder | PE, cognitive restructuring, relapse prevention, exposure techniques | CBT | Large improvements for panic and agoraphobia severity for iCBT compared to waitlist and information controls; similar results of iCBT and face-to-face CBT in reducing panic and agoraphobia symptoms; large within-group improvements for panic, medium for agoraphobia symptom severity; quality of evidence: low-moderate (assessed using the Cochrane risk-of-bias tool and ROBINS-I tool) | No impact of program length, inclusion or arousal reduction techniques, degree of clinician support | Not reported | Not reported | Not reported |  |
| Rees et al. (2015) ^83^ | No | Participants with anxiety disorders (eg, PTSD, obsessive-compulsive, social phobia) and/or depression | Treatment components based on CBT principles delivered using video conferencing software | CBT | Treatment effective in reducing anxiety symptoms (moderate – large effect sizes), comparable to face-to-face treatment; quality of evidence: low (eg, partly no controls or case studies, small sample sizes), no standardized approach was used to assess the quality of included studies | Lack of comparison with active control groups | Not reported | Not reported | Cost effectiveness reported; no data on savings provided |  |
| Richardson et al. (2010) ^60^ | No | Children and adolescents with depression | Stressbusters; Master your Mood online (group therapy via online chat room), Catch It (CBT principles with aspects of interpersonal therapy and behavioral activation); MoodGym (CBT- based, delivered online); iCBT: Cool Teens, BRAVE; including intervention components such as PE, quizzes, homework assignments, case vignettes, narration, cartoons, educational and training videos | CBT | 30% - 78% of included participants no longer met diagnostic criteria for primary diagnoses; improvements in depressive symptoms only in participants completing ≥3 sessions; quality of evidence: low (eg, 50% case studies, studies without control groups, possibility of publication bias, small number of databases), no standardized approach was used to assess the quality of included studies | Reduction in clinical symptoms and improvements in behavior, self-esteem and cognitions in all studies; preliminary evidence for ICBT as acceptable and effective for children/ adolescents | Moderate-high satisfaction with treatment from children and parents despite often high levels of drop out and non-completion | Not reported | Not reported |  |
| Rost et al. (2017) ^41^ | No | Individuals with depression | Mindfulness-based cognitive therapy, behavioral activation, MI | CBT | Effectiveness of digital interventions on depressive symptoms; drop-out rates comparable to face-to-face treatment; quality of evidence: low (eg, high heterogeneity, possibility of biases), no standardized approach was used to assess the quality of included studies due to high variability in study type | Not reported | High level of acceptance | Not reported | Not reported |  |
| Pasarelu et al. (2017) ^66^ | Yes | Nonclinical and clinical population (depressive and anxiety symptoms) | iCBT: transdiagnostic/ tailored, clinician guided with interview/ self-guided with interview/ self-guided, 4-25 modules | CBT | Medium-large effect for iCBT on anxiety and depression outcomes; medium effect on QoL; large effect on generic outcome measures; no differences on anxiety and QoL compared to disorder-specific treatments; quality of evidence: moderate to high (assessed using the Cochrane risk-of-bias tool) | Moderate effect on comorbidities; differences in depression outcomes compared to disorder-specific; treatment small-moderate heterogeneity | Not reported | Not reported | Not reported |  |
| Pittock et al. (2018) ^77^ | No | Patients with symptoms of bulimia nervosa | Weekly emails, F, tasks, manuals, email support, messaging with coach, face-to-face evaluations, group online setting | CBT | Large effects for iCBT in binge eating and purging reduction; sustained at follow-up; no overall significant superiority to controls; quality of evidence: moderate (assessed using the Cochrane risk-of-bias tool) | Not reported | Not reported | Not reported | Not reported |  |
| Massoudi et al. (2019) ^51^ | Yes | Patients in primary care with depressive/ anxiety symptoms/ disorders | Monitoring, PE, relaxation, behavioral activation, intensive disease management, mindfulness, ACT, medication management, assessment of symptoms, email reminder | CBT, Transtheoretical Model of Behavioral Change | Small effect of e-health interventions for depression compared to control groups/TAS, moderate effect compared to waitlist; effects maintained in long-term; no evidence for effectiveness for anxiety; quality of evidence: low-moderate (assessed using the Cochrane risk-of-bias tool) | Internet-based interventions as useful and acceptable for users as common treatment | Not reported | Not reported | Guided and self-guided e-health interventions more cost-effective than TAU; usual primary care provision for depression more cost-effective than guided e-health interventions and primary care; guided e-health intervention 94% likely to be cost-effective compared to waitlists; no data on savings provided |  |
| Lewis et al. (2019) ^85^ | Yes | Adults with PTSD | PTSD-Coach, trauma-focused PE, skills training, imaginal exposure, cognitive restructuring | CBT | Clinically relevant reduction in PTSD symptoms as compared to waiting list control; quality of evidence: low (assessed using Cochrane risk-of-bias tool) | No evidence to support maintenance of symptom improvement at follow-up (3–6 months) | Not reported | Not reported | Not reported |  |
| Harrer et al. (2019) ^38^ | Yes | University students with symptoms of depression, anxiety, stress, sleep problems, and eating disorders | Mindfulness based intervention, Talk to Me, eating disorder prevention, MoodGym, problem solving simulator, PE, present control intervention, eating and stress management, ACT based intervention, cognitive bias modification | CBT, CBM, third wave CBT, Emotional Disclosure, Skills Training, Personalized F | Small effect on depression, stress and anxiety; moderate effect on eating disorder symptoms and role functioning; no significant effect on well‐being; quality of evidence: moderate, high risk of bias in 50% of studies (assessed using the Cochrane risk-of-bias tool) | Not reported | Not reported | Not reported | Not reported |  |
| Hedman et al. (2012) ^113^ | No | Adult patients (eg, with depression, social phobia, panic disorder, PTSD) | Online bibliotherapy, therapist contact over the internet (text messages, email), PE | CBT | Large effect sizes on reducing depression, panic disorder, social phobia, small-moderate effect sizes on lowering chronic pain; quality of evidence: high for depression, panic disorder and social phobia (assessed with APA criteria for evidence) | ICBT tested for 25 different clinical disorders; ICBT produces equivalent effects compared to conventional CBT | Not reported | Not reported | 57% average probability of ICBT being cost-effective at willingness to pay of zero; cost-effective intervention compared with no treatment |  |
| Grist et al. (2019) ^63^ | Yes | Children and adolescents with anxiety and depression symptoms | films, text, animations, interactive fantasy game, computerized spider exposure therapy, interactive presentation, workbook, self-help manual, anxiety management, dot probe task, problem solving | CBT, computer-delivered Attention Bias Modification programs (ABM), Cognitive Bias Modification programs (CBM), video games utilizing Neurofeedback, Biofeedback, ER Training, internet-based ACT program, Problem-Solving Therapy | Small effect of technology delivered interventions compared to waitlist; CBT medium effect, ABM small effect; no significant benefit over control groups; quality of evidence: high risk of detection bias, low risk of attrition bias (assessed using the Cochrane risk-of-bias tool) | CBT based technology delivered interventions are useful when access to traditional psychotherapies is limited/delayed; type of control condition, problem severity, therapeutic support, parental support, and continuation of other ongoing treatment as moderators | Engagement was improved by therapeutic support | Not reported | Low cost alternative treatment when face to face treatments are not available or feasible; no data on savings provided |  |
| Davies et al. (2014) ^36^ | Yes | University students with symptoms of depression, anxiety, psychological distress | Stress management, improving relationship functioning, decreasing elevated levels of perfectionism, mindfulness, social support, increasing use of lucid dreaming, | CBT, Mindfulness, Stress Management Theory, Cognitive Learning Theory, Lucid Dreaming | Improvements in anxiety, depression, stress compared to inactive control; no support for either condition for anxiety or depression compared to active controls; quality of evidence: low (eg, skewed data), moderate risk of bias (assessed using the Cochrane risk-of-bias tool) | Not reported | Interventions are highly useable, satisfactory, and perceived as moderately to highly useful and helpful | Not reported | Not reported |  |
| Coughtrey et al. (2018) ^82^ | No | Population with depression and other medical conditions (eg, HIV/AIDS, multiple sclerosis, Parkinson’s disease) | Telephone-based Interventions, cognitive and behavioral components | CBT, Interpersonal Psychotherapy, Behavioral Activation, Exposure and Response Prevention, Applied Relaxation | Small to high effects on reducing symptoms of depression or anxiety (greater for uncontrolled compared to controlled studies); but significant change in only 4/14 studies; quality of evidence: moderate to high (assessed using the Effective Public Health Practice Project Quality Assessment Tool) | Not reported | Not reported | Not reported | Not reported |  |
| Asuzu et al. (2019) ^58^ | Yes | Adolescents and young adults (<24 years) with cannabis misuse | Cannabis specific and nonspecific; prevention program tailored to mother-daughter-dyad, RealTeen, Climate Schools, Computer brief intervention | Family Interaction Theory; Personalized and Corrective Normative F; Harm Reduction; Social Competency and Skills Building; Motivational Enhancement Model | Small-medium effects in reduction of cannabis use (7/11); quality of evidence: low (assessed using the National Institute of Health quality rating tool) | e-Health can overcome barriers to access care; longer duration of intervention adherence | Not reported | Not reported | Not reported |  |
| Baker et al. (2018) ^86^ | Yes | Adults with psychotic disorders | Telephone Intervention: relapse prevention, medication adherence, reduction of smoking and cardiovascular disease risk behaviors | Not reported; person-delivered interventions (spoken word and psychological strategies) | Relapse prevention (n=5/8), medication adherence (n=1/3); at least half of outcomes in favor of the telephone intervention, comparable levels of improvement; quality of evidence: low (eg, high variation in quality, many uncontrolled studies), no standardized approach was used to assess the quality of included studies | Telephone interventions were feasible and effective for improving health outcomes | Not reported | Not reported | Not reported |  |
| Chebli et al. (2016) ^53^ | No | Pathological gamblers, problem drinkers, smokers, opioid dependent outpatients | Support calls via telephone (positive F, encouragement, answer questions), SMS, email prompts, voice respond messages; peer based social support; behavioral approaches, internet-based modules/ assignments, face-to-face meetings, internet-based forum, information, self-help kit | CBT, MI | Consistent evidence for positive treatment outcomes for addictive behavior (effect size not reported); positive behavioral changes through reduction of problematic behaviors; quality of evidence: moderate (assessed using the Downs and Black checklist) | Reduces barriers of access | High satisfaction with SC intervention; preference for Internet-based service in opioid users | Not reported | Time-efficient and cost-effective, compared to face-to face  treatment methods; no data on savings provided |  |
| Danielsson et al. (2014) ^74^ | Yes | Adults with substance misuse (ie, cigarettes, alcohol) and/or pathological gambling | Eg, Online SC training, digital multimedia intervention, emails, SMS; Gambling & Alcohol misuse: Internet intervention with emails, telephone helplines, drinking journal, decision making modules, individual/group intervention, self-help booklet, craving helpline | MI, F, PE, CBT | Telephone helplines can reduce tobacco smoking; inconsistent effects for alcohol use and gambling; all together inconsistent evidence of eHealth on tobacco/ alcohol/ gambling; quality of evidence: low (eg, lack of controls, very high attrition rates), no standardized approach was used to assess the quality of included studies | Lack of studies with control groups | Not reported | Not reported | Not reported |  |
| Gilmore et al. (2017) ^76^ | No | Patients with co-occurring PTSD/ trauma symptoms and substance use disorder | Web-based modules, telehealth treatment, skill training, symptom management, MI, expert advice | Behavioral therapy | eHealth efficacious in reducing substance use and trauma symptoms: significant decrease in trauma symptoms in 3/4 studies; significant decrease in substance use in 4/6 studies; quality of evidence: moderate-high (assessed using the GRADE approach) | Technology-based interventions are feasible; moderate-high quality of studies | Not reported | Not reported | Not reported |  |
| *Notes:* CBT= Cognitive Behavioral Therapy; PE=psychoeducation; F=feedback; SC=smoking cessation; TAU=treatment as usual; VTC=video teleconferencing. | | | | | | | | | | |

**Table S2**. Included studies on mHealth interventions

| Reference | Meta-analyses | Population | Intervention components | Theoretical and evidence base on process/outcomes | Primary outcome and quality of evidence | Secondary outcomes | Quality from user perspective | Safety | Cost effectiveness |
| --- | --- | --- | --- | --- | --- | --- | --- | --- | --- |
| Mental Health Promotion and Prevention | | | | | | | | | |
| Sucala et al. (2016) ^110^ | No | Not reported | Meditation, breathing exercises, digital diary, cognitive restructuring, emotional ratings, problem solving, rational statements, goal setting, hypnosis, physical exercise | No information for 63,5% of the apps, 26,9% with Cognitive Behavioral Approach, 7,7% using mixed approach | Great majority of the mHealth apps do not offer evidence-based interventions; quality of evidence: low (eg, no information on evidence-base in 96,2% of the apps; none of the 14 apps reported studies on effectiveness); no standardized approach was used to assess the quality of included studies/apps | Only 3,8% of the apps have been rigorously tested | Not reported | Possible damaging consequences for people suffering from anxiety, who may use untested apps instead of specialized care | Not reported |
| Edwards et al. (2016) ^114^ | No | Not reported | Gamification: F, monitoring, reward and threat, goals and planning; individual techniques: self-monitoring of behavior, nonspecific reward, social support unspecified, nonspecific incentive and focus on past success | Behavior Change Technique | Median number of techniques per app was 14; Common combinations: goal setting, self-monitoring,  nonspecific reward and nonspecific incentive; goal setting, self-monitoring and focus on past success; no correlation between number of techniques and user ratings or price; no standardized approach was used to assess the quality of included studies/apps | Few health apps employ gamification: wide variation in the use of behavior change techniques, which may limit potential to improve health outcomes; no correlation between user rating (possible proxy for health benefits) and game content or price | Not reported | Not reported | Not reported |
| Böhm et al. (2019) ^90^ | No | Healthy Children and adolescents (aged 6-18 years) | F, self-monitoring, goal setting, strategies to overcome barriers, information | Social Cognitive Theory, Behavior Change Technique, | No statistically significant effects on PA-related outcomes with mHealth tools; quality of evidence: low (eg, small number of studies, inadequate use of validated measures, missing RCTs, heterogeneity of interventions); no standardized approach was used to assess the quality of included apps/studies | No effect of activity trackers on PA | 81% interested in trying various PA-apps, 92% enjoyed requirement of being active | Not reported | Not reported |
| Kim et al. (2019) ^93^ | Yes | Adults (eg, overweight university staff and students) | Lifestyle intervention, education and coaching to promote PA, dietary counseling, education related to health behavior, F | Transtheoretical  model, Theory of Planned Behavior | Significant increase in PA, significant weight loss in the intervention groups; quality of evidence: moderate (low risk of bias assessed using the Cochrane risk-of-bias tool), but only 5 studies included | Smartphone-based health interventions significantly affect weight loss and increase PA; modest evidence for using smartphone health programs to improve young adults' PA, weight control and body mass index | Not reported | Not reported | Not reported |
| Song et al. (2019) ^102^ | No | General population with unhealthy alcohol use | Motivation (encouraging messages, peer support, monetary compensation), general and personalized information, reminder, and warning | Behavioral change theories (eg, Theory of Planned Behavior, Health Belief Model, Theory of Reasoned Action, Social Learning, Theory) | 63% mHealth interventions brought significant positive outcomes in improving participants’ health compared to traditional methods; quality of evidence: high (assessed using Mixed Methods Appraisal Tool) | 33% cognitive changes significantly improved | Not reported | Significant negative outcome reported for male participants in intervention arm in one study | Cost effectiveness reported; no data on savings provided |
| Feter et al. (2019) ^92^ | Yes | Not reported | PA interventions with SMS or App promotion using accelerometer, pedometer, questionnaire, daily diary, | Not reported | Efficient in increasing minutes and steps per day in adults when compared to baseline; quality of evidence: moderate-high (assessed using the Downs and Black scale) | Mobile phone-based PA interventions were effective to increase minutes and steps per day in adults; promotion of engagement through curiosity | Not reported | Not reported | Not reported |
| Bort-Roig et al. (2014) ^91^ | No | General population, special populations (eg, obese patients) | Smartphone strategies to influence PA; PA profiles, goal setting, real-time F, social support networking, and online expert consultation | Behavior Change Theories, including Social Cognitive Theory and the Trans-theoretical Model | PA increases and one study reported PA maintenance over 3 months; quality of evidence: low (eg, small sample sizes, short study periods), no standardized approach was used to assess the quality of included studies | Validity of phone-based assessment rarely considered; measurement properties found average-to-excellent levels of accuracy for different behaviors; smartphone apps have potential for PA promotion | PA profiles, real-time F, social networking, expert consultation, and goal setting were identified as key features that facilitated PA engagement | Not reported | Not reported |
| Muntaner et al. (2016) ^95^ | No | General population | Monitoring, F, information, interactive voice response system, questionnaire, messages with helpful hints, goal setting, motivational messages, reminders | Social Cognitive Theory, Protection Motivation Theory, Transtheoretical Model, Theory of Planned Behavior, Goal Setting Theory, Problem Solving Theory | 6/12 studies reported significant increases in PA levels; quality of evidence: low-moderate (eg, lack of information provided by studies) (assessed using the Downs and Black scale) | Important tool for disease prevention and interventions affecting health behavior | Not reported | Important to evaluate safety and effectiveness before launching an app | Mobile devices are inexpensive tools; no data on savings provided |
| Rathbone et al. (2017) ^99^ | No | General population | Self-monitoring, SMS, PE, podcast, journal | CBT | Significant decrease in anxiety and stress with medium to large effect sizes, quality of evidence: low to moderate (assessed using Cochrane risk-of-bias tool); 8 of the studies without control groups | Significant increase in step count, moderate effects on moods, moderate to large effects on depression, large effect on increased positivity | High satisfaction with texting intervention, individuals perceive mHealth to be effective | Not reported | Cost effectiveness reported; no data on savings provided |
| Alyami et al. (2017) ^29^ | No | Not reported | PE, symptom management, therapeutic treatment, self-assessment, supportive resources, multi-purpose | CBT, mobile-based Interpersonal Psychotherapy, smartphone-based CBM for Attention | Over 60% of apps exclusively focused on social anxiety, remainder targeted social anxiety and related conditions; most developers did not provide information on organizational affiliations or content source; most apps used multimedia while 17 apps used text only; quality of evidence not reported | Social anxiety apps have potential to overcome barriers to accessing treatment; none of the apps identified have had studies on their effectiveness published; evidence base is lacking, currently not possible to recommend their use | Not reported | Not reported | Not reported |
| Treatment | | | | | | | | | |
| Sander et al. (2020) ^109^ | No | Soldiers or veterans, family members (of people with PTSD, Clinicians, Children, Police officers or public safety professionals) | 63.8% of apps offered elements of  mindfulness, relaxation, breathing, or body exercises; information/PE, assessment, monitoring and tracking, F, skill training, exposure, mindfulness, relaxation, breathing, body exercises, resource orientation, tips and advices | CBT, Behavior Therapy, Systemic Therapy, third wave CBT, Psychodynamic therapy | Overall app quality based on the MARS was medium. Most offered a wide range of content, including established psychological PTSD treatment methods (processing of trauma-related emotions and beliefs, relaxation exercises, and PE); quality of evidence not reported | Users are confronted with great difficulties in identifying useful high-quality apps, most apps lack an evidence-base | Not reported | Inadequate data protection and privacy declarations: passwords and logins were required in only 10% of apps, 17% provided a privacy statement | Not reported |
| Terhorst et al. (2018) ^111^ | No | Patients with depression | Assessment, PE, monitoring and tracking | third wave CBT, CBT, Behavioral Therapy, Alternative Medicine | Overall quality was average. Four high-quality apps were identified and recommended with reservations for practical use; quality of evidence not reported | Depression apps available in German showed an average quality; general lack of evidence; identified apps can only be recommended with reservations | Not reported | Lack of evidence | Not reported |
| Ilagan et al. (2020) ^108^ | Yes | Adults with borderline personality disorder (BPD) | Positive stimuli paired with self, personalized safety-plan, PE on suicidal thoughts, symptom monitoring, digital hope kit, self-help skills, anger management exercises, mindfulness meditation exercises | CBT | No significant effect of apps on BPD symptoms and general psychopathology, quality of evidence: moderate (assessed using Cochrane Collaboration Risk of Bias Assessment Tool) | Evidence on BPD-related interventions delivered via smart- phone apps is still weak; too early to recommend them | Dropout rates ranging from 0 – 56,7%; smartphone applications reported to be user-friendly | Some studies reported suicide attempts or slower reduction of suicide risk compared to control group | Not reported |
| Loo Gee et al. (2016) ^97^ | No | General population and patients (aged 17-55 years) | Integrative EMIs: self-monitoring of symptoms, delivery of automated or therapist-delivered psychotherapy content | CBT | EMIs may be a promising treatment for generalized anxiety and may be effective for reducing stress; quality of evidence: low-moderate (assessed using the Cochrane Effective Practice and Organization of Care  Group criteria) | EMIs are associated with a small, but significant reduction in generalized anxiety symptoms; EMIs targeting stress may be effective; few studies examined EMIs targeting other anxiety-related conditions: mixed results | Not reported | Not reported | Not reported |
| Rootes-Murdy et al. (2018) ^100^ | No | Patients with mood disorders | Apps, phone calls, SMS, mobile web-based surveys; electronic pill dispenser, face-to-face conversation with animated agent | Not reported | Overall satisfaction and feasibility of mobile technology, reduction in mood symptoms; few examined effectiveness of mobile technology improving medication adherence through RCTs; results represent approximately 10% higher mean medication adherence rates when compared to observational studies; quality of evidence: low (eg, mostly observational studies, technologies only used for short period), no standardized approach was used to assess the quality of included studies | Mobile technologies have potential to improve medication adherence, can be utilized for symptom tracking, side effects tracking, direct links to prescription refills, and provide patients with greater ownership over their treatment progress | Improvements in medication adherence could not be conclusively attributed to intervention due to the observational study designs and lack of comparison groups | Not reported | Not reported |
| Miralles et al. (2020) ^25^ | No | Not reported | Psychological interventions for mental disorders delivered via smartphone, plus SMS and phone calls | CBT, Behavior Therapy, third wave CBT | 72.7% of the papers focused on six mental disorders: depression, anxiety, trauma and stressor-related, substance-related and addiction, schizophrenia spectrum, and other psychotic disorders, or a combination of disorders; quality of evidence: low (eg, little percentage of RCTs, few studies asses effect of mHealth on symptomatology), no standardized approach was used to assess the quality of included studies | Depression and anxiety disorders are primarily covered, in line with their real-world prevalence | Not reported | 31%-49% of included mental health apps do not include a privacy policy; data can be distributed to (third-party) services for storage and analysis; risk that data is being transmitted over insecure networks | Not reported |

*Notes:* PA=physical activity; F=feedback; EMIs=ecological momentary intervention.

**Table S3.** Summary of included reviews on interventions including eHealth and mHealth interventions (mixed)

| Reference | Meta-analyses | Population | Intervention components | Theoretical and evidence base on process/outcomes | Primary outcomes and quality of evidence | Secondary outcomes | Quality from user perspective | Safety | Cost effectiveness |  |  |  |
| --- | --- | --- | --- | --- | --- | --- | --- | --- | --- | --- | --- | --- |
| Mental Health Promotion and Prevention | | | | | | | | | |  |  |  |
|  |  |  |  |  |  |  |  |  |  |  |  |  |
| Rose et al. (2017) ^94^ | No | General population of adolescents (aged 10-19 years); specific at-risk populations | Diet and PA education, goal setting, monitoring, parental involvement; counselling; health information, assessment of behavior, skill building, reward system, F, pedometer, self-monitoring, cycling video game, fruit promotion via email, family involvement | Not reported | Significant improvements in behavior in 8/22; significant improvement in diet and/or PA for majority of interventions with goal setting; self-monitoring less effective without goal setting; significant improvement in behavior in most interventions with family involvement; quality of evidence: low-moderate (eg, poor handling of confounding factors, participant selection bias), assessed using a checklist based on quality assessment criteria by the Centre for Reviews and Dissemination | Lack of evidence on medium to long term effects | Not reported | Not reported | Not reported |  |  |  |
| Baker et al. (2018) ^64^ | No | Older adults | ICT based interventions: using devices with touchscreen, social network services | Social concepts often poorly defined | No evaluation of efficacy due to insufficient attention to social concepts; quality of evidence: low (eg, very small sample sizes, poorly defined outcomes), Multiple tools were used to assess the quality of included studies (ie, Cochrane Collaboration Risk of Bias Assessment Tool; PEDro scale, Downs and Black scale) | Poorly defined social outcomes; limited methodologies to evaluate interventions | Not reported | Not reported | Not reported |  |  |  |
| Kreuze et al. (2017) ^106^ | No | Heterogeneous, including adults, adolescents, university students | Virtual Hope Box (support, comfort, distract or relax using audio, video, pictures, games, mindfulness exercises, messages, inspirational quotes and coping statements), coping strategies, behavioral activation, interpersonal psychotherapy, community resiliency concepts, problem solving, F | CBT, Cognitive Therapy, Dialectic Behavior Therapy, Mindfulness-based Cognitive Therapy, Problem-solving Therapy | Participants  improved significantly on depression, anxiety, hopelessness, self-esteem, and negative automatic  thoughts; promising evidence for reduction of suicidal ideation and mental health co-morbidities; quality of evidence: low (eg, unprecise study effects, small sample sizes, low engagement); no standardized approach was used to assess the quality of included studies | Not reported | Not reported | Elevated suicide risk at follow-up | The mean  incremental cost-effectiveness ratio was US$37,985; willingness  to pay for a favorable treatment response was  high |  |  |  |
| Lau et al. (2011) ^34^ | No | Children and adolescents | Goal setting, tips, comic stories, social support emails, self-monitoring, quizzes, games, charts to plan activities, motivational reminder, F, tailored information, online counselling | Health Behavior Change Theory, Social Cognitive Theory, Transtheoretical Model, Relapse Prevention Model | Evidence supporting efficacy of interventions on improving psychosocial outcomes vs. conventional or no treatment, less consistent for behavioral outcomes; quality of evidence: 7/9 studies good methodological quality; assessed using the Cochrane risk-of-bias tool | Not reported | Not reported | Not reported | Not reported |  |  |  |
| Yonker et al. (2015) ^103^ | No | Adolescents and young adults (aged 11-25 years) | Observation, providing health information, engaging in community, recruiting research participants | Not reported | Positive impact on mental health outcomes; mixed results for community engagement; quality of evidence: low (eg, 62% of studies with sampling bias, 24% with incomplete datasets, 19% with small sample size); no standardized approach was used to assess the quality of included studies | 75 of included studies were observational, only 12 including an intervention | One study found that adolescents did not feel comfortable having an unknown health care provider screen their social media accounts | Privacy and confidentiality issues | Cost effective recruitment strategy; no data on savings provided |  |  |  |
| Treatment |  |  |  |  |  |  |  |  |  |  |  |  |
|  |  |  |  |  |  |  |  |  |  |  |  |  |
| Berry et al. (2016) ^115^ | No | Individuals with severe mental health problems (eg, bipolar disorder, schizoaffective disorder, psychotic disorder, schizophrenia) | Medication reminders, information, advice, helpline for crisis intervention, decision making tools, coping skills, check-ins | CBT | High acceptability of online and mobile phone-delivered interventions; acceptability was higher for interventions delivered via mobile phones as compared to online formats; quality of evidence: low (eg, varied findings, limited number of studies, inaccurate measurement of acceptability), no standardized approach was used to assess the quality of included studies | No significant relationship between clinical characteristics and acceptability | Many participants satisfied with clarity and appearance of interventions | Concerns about safety, privacy and confidentiality in some studies | Not reported |  |  |  |
| Walsh et al. (2016) ^47^ | No | Population with variety of mental illnesses eg, anxiety, depression, bipolar disorder, eating disorder | Technology based symptom-monitoring, F | Self-monitoring, CBT | Acceptability of monitoring is related to perceived validity, ease of practice, convenient technology, appropriate frequency, helpfulness of F, impact of monitoring on participants’ ability to manage health and personal relationships; quality of evidence: low (eg, descriptive results, high heterogeneity), no standardized approach was used to assess the quality of included studies due to low quality of data | Not reported | Moderate-strong rates of participation in 2/3 of studies, lower rates in 1/3; overall positive experience; perceived lack of support for unguided participants (F messages unhelpful) | Not reported | Not reported |  |  |  |
| Rice et al. (2014) ^69^ | No | Young people with depression | PE, behavioral activation, thought monitoring, skills training, online support group postings | CBT | Most intervention studies demonstrated superiority of online intervention vs. comparison treatment (treatment as usual, waitlist, brief PE); no standardized approach was used to assess the quality of included studies | There are young individuals who may be more willing to disclose information digitally than in person | Some interventions report relatively low levels of engagement | Privacy concerns reported | Cost effectiveness reported; no data on savings provided |  |  |  |
| Aref-Adib et al. (2019) ^43^ | No | Patients with schizophreniform disorders and/or bipolar disorder | Web-based computer program, Telecare, online platform, mHealth, peer-run web-based computer program; eg, mindfulness, shared decision making, aid to build relationships and to communicate. | Not reported | Factors that affect implementation: eg, lack of motivation, poor information technology skills, language problems, poor mental state, labor-intensive scheduling, availability of telehealth space, equipment, attitudes/beliefs about digital interventions; quality of evidence: low, no standardized approach was used to assess the quality of included studies as research is in early stages | No single factor identified as key barrier/facilitator; majority of factors centered at level of individual or intervention; complexity of digital interventions as barrier for people with psychiatric symptoms, low premorbid intelligence quotient, or low IT skills | Easy and flexible access to digital interventions; possibility of sharing intervention with others | A minority of people with psychosis became paranoid or experienced an increase in symptoms | Costs for developing interventions and delivery as barriers; no data on savings provided |  |  |  |
| Biagianti et al. (2018) ^42^ | No | Adults with psychotic disorder | Internet peer-support, peer-support bulletin board, PE, therapy groups, moderated peer discussion forums, psychosocial interventions, social networking, SMS based motivational coaching, goal setting, computerized social cognition training, group texting | Social Cognition Training | Digital interventions+ peer-to-peer communication associated with good retention rates (78,4%), + mental health providers improved engagement, peer-to-peer communication highly engaging; quality of evidence: low (eg, small samples, no control groups); no standardized approach was used to assess the quality of the included studies | All interventions feasible and acceptable for broad spectrum of mental health problems though significant variation in their effects on health-related outcomes has been found | Acceptability high for online social networking integrated in evidence-based therapies | Increased anxiety, low self-esteem, psychological distress, and depression in young people with frequent social media use | Cost effective; no data on savings provided |  |  |  |
| Faurholt-Jepsen et al. (2016) ^45^ | No | In- and outpatients with bipolar disorder type 1 | Computer, ChronoRecord, PDA, Prism, Smartphone, MONARCA, Personal Life Chart App, Paper Pencil, Moodswings, Websites on healthy lifestyle; self-monitoring of mood, medications, self-management, PE, F | CBT | Consistent evidence for validity of electronic self-monitored mood for depression; no consistent evidence for validity of electronic self-monitored mood for mania; quality of evidence: moderate (eg, possible risks of biases) (assessed using the Cochrane risk-of-bias tool) | Not reported | Not reported | Not reported | Not reported |  |  |  |
| Goldberg et al., (2018) ^44^ | No | Patients with mental disorders (eg, depression, bulimia, psychotic disorder, substance use, bipolar disorder) | Remote measurement-based care as part of treatment eg, biofeedback, self-guided intervention, psychotherapy, remote therapist support | Remote measurement-based care (RMBC) | Positive effects of the intervention for RMBC as part of multicomponent intervention; inconsistent evidence for clinical effectiveness of intervention alone (effective in 1/3 studies, but not as effective as part of multicomponent intervention); quality of evidence: low (meta-analysis could not be conducted due to high heterogeneity of outcomes and study design), no standardized approach was used to assess the quality of included studies | Not reported | Moderate-high adherence/acceptability for daily, weekly and monthly assessment with tendency to higher adherence for less frequent assessment; moderate-high satisfaction with RMBC as stand-alone assessment and part of a multicomponent intervention | Not reported | Not reported |  |  |  |
| Perry et al. (2016) ^107^ | No | Young people (aged 12 – 25 years) with suicidal thoughts | Cognitive restructuring, behavioral activation, focus on problem solving around suicidal ideation, video diaries, message board | CBT | Significant reductions in suicidality, depression and hopelessness (small-moderate effect sizes); quality of evidence: low (only one study, small sample, high attrition rate, no control group); no standardized approach was used to assess the quality of the included studies | Not reported | Not reported | Potential risk of cyber-bullying, unknown safety of vulnerable information | Potential cost reduction; no data on savings provided |  |  |  |
| Wright et al. (2019) ^71^ | Yes | Population (≥16 years) with diagnosed depression | Multimedia, text (with images) via computer or app | CBT | Small to moderate effects of ICBT compared to control conditions; significantly larger effects for studies with support from clinician (moderate effect) than for studies without support (small effect); quality of evidence: moderate-high (assessed using the CLEAR NPT) | Lower effect sizes for studies with lower completion rates and studies in primary care practices | Not reported | Not reported | Not reported |  |  |  |
| Xiang et al. (2019) ^73^ | No | Older adults (≥50 years) with symptoms or diagnosis of depression | Therapist- or self-guided CBT: Beating the Blues, Sadness Program, Manage your Mood, Wellbeing Plus Course, MoodTech, | CBT | Large within-group and between-group effect sizes; quality of evidence: low (assessed using the Cochrane risk-of-bias tool) | Participant's age as moderator of effect size (negative correlation); larger effects for studies excluding participants with severe symptomatology | Not reported | Not reported | Not reported |  |  |  |
| Alvarez-Jimenes et al. (2014) ^40^ | No | ≥ 80% of participants diagnosed with schizophrenia-spectrum disorders | Web-based PE; moderated forums for patients and supporters; integrated web-based therapy, social networking and peer and expert moderation; personalized advice based on clinical monitoring; text messaging interventions | CBT | Efficient use in about 80% of patients; high perception as positive and useful, little dropout in follow-up (≤30%); quality of evidence: low (eg, poor description of methodology/results) (assessed using the Cochrane risk-of-bias tool) | Online and mobile-based interventions can improve positive psychotic symptoms, hospital admissions, socialization, social connectedness, depression, medication adherence | Feasible and acceptable to patients with schizophrenia (80% completed the majority of sessions); 26% had difficulties with online PE, 12% found online CBT not helpful | Need of careful evaluation of new interventions (engagement rates, intervention/patient factors) | Not reported |  |  |  |
| Arshad et al. (2020) ^104^ | Yes | Adults with suicide attempts/ meeting criteria for NSSI disorder, PTSD, depression, suicidal ideation | Dialectical behavioral therapy techniques, individual ER therapy, toolbox of support (eg, coping skills, strategies, crisis support) | CBT, DBT, individual ER therapy | Limited evidence for efficacy of internet- and mobile-based interventions for self-injurious thoughts and behaviors, reductions in SB in single-arm noncontrolled studies; beneficial effect on suicidal ideation compared to TAU, not when compared to active controls; quality of evidence: low (assessed using the Cochrane risk-of-bias tool) | Not reported | Text message-based services: high perceived helpfulness, good way to stay in touch with services; mobile phone apps: high perceived helpfulness and satisfaction with content;  internet-delivered CBT and DBT approaches: high perceived helpfulness and utility | Not reported | Not reported |  |  |  |
| Domhardt et al. (2020) ^26^ | Yes | Children and adolescents with mental and somatic disorders | Internet- and mobile based Interventions (IMIs): webpages, email, chat, videoconferencing, mobile app, instant messaging; psychotherapeutic orientation | CBT | Large effect for IMIs across mental disorders, medium effect of IMIs for somatic conditions compared to nonactive controls; quality of evidence: moderate (assessed using the AMSTAR-2 checklist) | Age, symptom severity and source of outcome assessment as moderators | Not reported | Not reported | Not reported |  |  |  |
| Meyer et al. (2018) ^78^ | Yes | Population with major depressive disorder, borderline personality disorder, variety of diagnoses | Monitoring, adherence promotion, PE, self-management, relapse prevention | CBT | Larger RCTs showed beneficial effects on symptoms and functioning (effect size not reported); quality of evidence: low (eg, small sample sizes, unreliable differentiation between on- and offline available software), no standardized approach was used to assess the quality of included studies | No study focused on well-being of relatives; identified studies mainly target mood disorders | SMART: app perceived as useful and helpful for self-management, significant associations between clinician-rated mood symptoms and daily self-ratings; DBT-Coach: easy to use and helpful for ER | Not reported | Not reported |  |  |  |
| Ye et al. (2014) ^67^ | Yes | Children and adolescents with anxiety and/or depressions | Self-monitoring of moods, stress, alcohol and cannabis use, SMS and phone call support, online self-help sessions, email, SMS, phone calls, family/ group/ teacher support | CBT | Medium effect size in reducing anxiety symptom severity compared to waitlist control, increased remission rate; nonsignificant reduction of depression symptom severity; no difference in anxiety/ depression symptoms between internet-based intervention and face-to-face intervention; no superiority of usual care; quality of evidence: moderate-high (assessed using a modified version of Quality Assessment Tool for Quantitative  Studies) | Not reported | Not reported | Not reported | Not reported |  |  |  |
| O’Rourke et al. (2016) ^55^ | No | Hazardous young drinkers | Behavioral interventions; web-based, email, text messages, social network sites; personalized F, monitoring, social norms F (SNS), PE, improving knowledge, self-efficacy and awareness of social norms | Not reported | Interactive approaches (text messaging, email, SNS) significantly reduce frequency of drinking; personalized electronic F reduces alcohol consumption, frequency of binge drinking, and drinking in a nonrisky way; quality of evidence: moderate (assessed using the Cochrane risk-of-bias tool) | No impact of intervention length on effectiveness | Interactive approaches increase ease of engaging in intervention with high acceptability | Not reported | Not reported |  |  |  |
| Dick et al. (2019) ^101^ | No | Third level students | F, descriptive norm correction, self-affirmation manipulation, theory-based messages, implementation intention tasks, motivational brief intervention, messages based on social cognitive theory | Social Cognitive Theory, little to no detail about design and development process of the intervention | Reduction of substance misuse and initiation (effect size not reported); quality of evidence: low (assessed using the Quality Assessment Tool for Quantitative Studies by the Effective Public Health Practice Project) | Low study quality (lack of blinding, self-report measures) | Lack of engagement | Increase in substance misuse in one study | Not reported |  |  |  |
| Giroux et al. (2017) ^24^ | No | Adults with high school education and high risk or problematic alcohol/drug use | PE, F, Craving and relapse management, behavior change, alcohol use journal, control strategies, social skills learning, chat with therapist | CBT, Theory of Planned Behavior, Solution focused, Self-control, Relapse Prevention | Significant decrease in substance use in 3/4 of studies; long term (12-month follow-up, investigated by only 2 studies): effects maintained for women; quality of evidence: moderate (assessed using the Cochrane criteria checklist) | Online interventions are accepted by people in workforce who seek help for the first time | Not reported | Not reported | eHealth presents a better cost-efficacy ratio; online interventions have potential to cover large areas at low costs and reach populations that are harder to reach; no data on savings provided |  |  |  |

*Notes:* CBT=Cognitive Behavioural Therapy; DBT=Dialectical behaviour therapy; PE=psychoeducation; PA=physical activity; F=feedback; ER=emotion regulation.
